# Differential Biophysical Behaviors of Closely Related Strains of Salmonella

**DOI:** 10.3389/fmicb.2020.00302

**Published:** 2020-02-25

**Authors:** Yameng Liu, Mark A. Hayes

**Affiliations:** School of Molecular Sciences, Arizona State University, Tempe, AZ, United States

**Keywords:** dielectrophoresis, *Salmonella*, electrokinetics, label-free, microfluidics, serotype, *Cubana*, *Poona*

## Abstract

*Salmonella* is an important pathogen and is a world-wide threat to food safety and public health. Surveillance of serotypes and fundamental biological and biochemical studies are supported by a wide variety of established and emerging bioanalytical techniques. These include classic serotyping based on the *Kauffmann–White* nomenclature and the emerging whole genome sequencing strategy. Another emerging strategy is native whole cell biophysical characterization which has yet to be applied to *Salmonella*. However, this technique has been shown to provide high resolution differentiation of serotypes with several other paired strains of other microbes and pathogens. To demonstrate that biophysical characterization might be useful for Salmonella serotyping, the closely related strains sv. *Cubana* and sv. *Poona* were chosen for study. These two serovars were subjected to biophysical measurements on a dielectrophoresis-based microfluidic device that generated full differentiation of the unlabeled and native cells. They were differentiated by the ratio of electrophoretic (EP) to dielectrophoretic (DEP) mobilities. This differentiation factor is 2.7 ± 0.3 × 10^10^ V/m^2^ for sv. *Cubana*, versus 2.2 ± 0.3 × 10^10^ V/m^2^ for sv. *Poona*. This work shows for the first time the differentiation, concentration, and characterization of the *Salmonella* serotypes by exploiting their biophysical properties. It may lead to a less expensive and more decentralized new tool and method for microbiologists, complimenting and working in parallel with other characterization methods.

## Introduction

There is an increasing array of methods to characterize microorganisms from whole genome sequencing to traditional culturing strategies (Chiou et al., 2015; Ibrahim and Morin, 2018). For *Salmonella*, a common foodborne pathogen that can cause disease in humans, the characterization must allow tracking of the contamination source by using appropriate subtyping tools (Tang et al., 2019). The “gold standard” classifying subtle differences between salmonella strains is based on the *Kauffmann–White* nomenclature (Grimont and Weill, 2007), representing a traditional phenotyping method that is logistically challenging, as it requires the use of more than 150 specific antisera and well-trained personnel to interpret the results (Diep et al., 2019). One emerging and unproven strategy is to directly assess the biophysical characteristics of the native and unlabeled cells toward correlating their properties with specific serotypes. In this study, two closely related serovars based on the similar antigens indicated in the *Kauffmann–White* categorization scheme are tested and were differentiated in their native state with simple electric field interactions.

The common microbe *Salmonella* is thought to be responsible for 450 deaths, 23,000 hospitalizations, and 1.4 million illnesses each year in the United States (Bishop et al., 2011). The typical symptom is abdominal pain and is diagnosed as gastroenteritis, with severe infections becoming life threatening. Food safety incidents and recalls continue in recent years, mostly associated with processed products (Pillai and Ricke, 2002; Maciorowski et al., 2004; Park et al., 2008; Hanning et al., 2009), and other food commodities (e.g., meat products, eggs, and vegetables) (Greig and Ravel, 2009; Wu et al., 2017; Ricke et al., 2018; Tang et al., 2019). These occurrences necessitate accurate and relatively rapid subtyping tools for identifying the original source of contamination (Olaimat and Holley, 2012; Barco et al., 2013; Shi et al., 2015). *Salmonella* is a diverse pathogen and there are over 2500 *Salmonella* serotypes (2007 data), which have been described (Grimont and Weill, 2007). Of these, 99% of human isolates belong to the subspecies *Salmonella enterica* subsp. *enterica* (also described equivalently as “subspecies I”).

The immunoreactivities to O and H antigens of each isolate define the serotype, where a substantial diversity exists within the antigens. A cell surface lipopolysaccharide structure makes up the O antigen and typically consists of four to six sugars. The various specific antigens can differ by the linkages between sugars, covalent bonds between the units, or differences in the sugars themselves. These are divided into “O group antigens” (specific sugar configuration of the O antigen structure) and “ancillary O antigens” (additional carbohydrates). On the other hand, a proteinaceous antigen, flagellin or H antigen, is located on the flagellum in a filamentous portion. The core structural elements of these proteins which provide the filamentous structure, C′ and N′ termini, are conserved. The middle region of flagellin is exposed on the surface and is antigenically variable. Like many taxonomic and categorization schemes, those associated with *Salmonella* are evolving and therefore include modern and systematic definitions along with archaic terms still in common usage.

**FIGURE 1:**
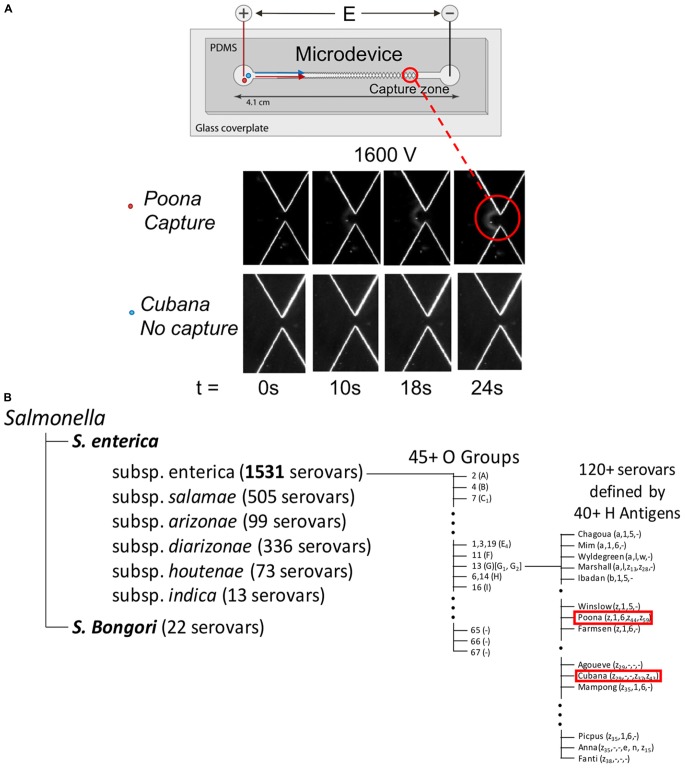
Graphic representation of microdevice **(A)** and *Kauffmann–White* nomenclature showing the relationship by phenotypic characterization between sv. *Cubana* and sv. *Poona* **(B)**. In part **(A)**, capture and concentration behavior of *Salmonella* serotypes in the DC-iDEP device. Insets images (bottom) of sv. *Cubana* and sv. *Poona* at 0, 10, 18, 24 s of 1600 V applied. The images were recorded at a 27-μm gate. With same potential (1600 V) applied, sv. *Poona* was captured but sv. *Cubana* was able to pass through. The graphic shown in part **(B)** indicates sv. *Cubana* and sv. *Poona* are closely associated with each other in comparison to the array of some 2500+ *Salmonella* serovars.

The two serotypes characterized in this study are *S. enterica* serotype *Cubana* and *S. enterica* serotype *Poona*. These strains belong to a group equivalently referred to as Group O:13 and Group G, and commonly Group O:13 (G) (Table 1 and Figure 1; Grimont and Weill, 2007). Noting that there are more than 1500 serovars (sv.) in *S. enterica* (Figure 1B), these serotypes are considered to be closely related with respect to the total breadth of serovars. Both of these serovars have been characterized with whole genome sequencing studies (Hoffmann et al., 2014; USFDA, 2014). This will provide a framework for assigning the quantified differences in biophysical properties presented here to specific biochemical origins.

**TABLE 1:** Listing of specific known antigens for two *Salmonella* strains using *Kauffmann–White* nomenclature.

Serotype	Group*	Somatic* (O) Antigen	Flagellar* (H) Antigen		
			Phase 1	Phase 2	Other
*Cubana*	O:13 (G)	1, 13, 23	z_29_	–	[z_37_] [z_34_]
*Poona*	O:13 (G)	1, 13, 22	z	1, 6	[z_44_] [z_59_]

*Notation from *Kauffmann–White* (Grimont and Weill, 2007). Underlined O factors are determined by phase conversion.

In this study, we demonstrate a rapid biophysical differentiation of two closely related strains of *Salmonella*, sv. *Cubana* and sv. *Poona*, using constant voltage gradient insulator-based dielectrophoresis (DC-iDEP) (Figure 1). The distinction is reflected by a different voltage at which each strain begins to capture, defining a specific characteristic and deterministic property for each strain. With some additional measurements, the specific forces regarding the electrokinetic (EK) and dielectrophoretic (DEP) mobilities are determined. These values allow some insights into the molecular and structural origins of the differentiation (Hilton and Hayes, 2019). This provides strong evidence that the simple measurement of the native and unlabeled cells may provide another valuable tool in the determination of serovars and basic science studies of *Salmonella.*

## Theory

The properties of particles dispersed in a buffer or solvent in the presence of an external electric field exhibit behavior directly according to their physical makeup. In this study, biophysical behaviors are identified within a DC-iDEP device by the applied voltage and location of cell capture. Particles in the device experience DEP and EK forces. Higher order electrophysical effects are described by DEP mobility (μ_DEP_). It can be expressed as (Jones, 1995; Nili and Green, 2014; Hilton and Hayes, 2019):

(1) $$\mu_{\text{DEP}}=\frac{\varepsilon_{\text{m}}\,r^{2}\,f_{\text{CM}}}{3\,\eta }$$

(2) $${\vec{\text{v}}}_{\text{DEP}}=\mu_{\text{DEP}}\nabla |\vec{E}{|}^{2}$$

where ε*_m_* is the permittivity of the medium, *r* is the radius of the particle, *f*_CM_ is the Clausius–Mossotti factor, and η is the medium viscosity. EK force is the combination of electrophoretic (EP) force (first order effects, monopole moment) and electroosmotic flow (EOF). Reflecting these processes, EK mobility (μ_EK_) is defined by

(3) $$\mu_{\text{EK}}=\mu_{\text{EP}}+\mu_{\text{EOF}}$$

where μ_EP_ is EP mobility and μ_EOF_ is the EOF mobility. And they can be described by

(4) $$\mu_{\text{EP}}=\frac{\varepsilon_{\text{m}}\,\zeta_{\text{p}}}{\eta }$$

(5) $$\mu_{\text{EOF}}=\frac{-\varepsilon_{\text{m}}\,\zeta_{\text{m}}}{\eta }$$

where ε_m_ is the permittivity of the medium, ζ*_p_* is the EK (zeta) potential of the particle, and ζ*_m_* is the EK (zeta) potential of the medium/wall system. The value of μ_EK_ was determined for both strains by particle tracking velocimetry (Crowther et al., 2019; Hilton and Hayes, 2019) at various applied voltages based on:

(6) $${\vec{\text{v}}}_{\text{EK}}=\mu_{\text{EK}}\vec{E}$$

where ${\vec{\text{v}}}_{\text{EK}}$ is the velocity of the particle in an open channel.

The capture of the particles can be observed when the flux of particles ($\vec{j}$) is zero in the channel with the condition:

(7) $$\vec{J}\cdot \vec{E}=0$$

(8) $$\left(\mu_{\text{EK}}E+\mu_{\text{DEP}}\nabla |\vec{E}{|}^{2}\right)\cdot \vec{E}>0$$

(9) $$\frac{\nabla |\vec{E}{|}^{2}}{E^{2}}\cdot \vec{E}\geq \frac{\mu_{\text{EK}}}{\mu_{\text{DEP}}}$$

where $\nabla |\vec{E}|$ is the gradient of the electric field and *E* and $\vec{E}$ are the scalar and vector electric field, respectively. The ratio of EK to DEP mobilities $\frac{\mu_{\text{EK}}}{\mu_{\text{DEP}}}$ (EKMr) which relates size, conductivity, surface charge, and other factors of the particle to the electric field properties are used to distinguish the subtle differences between the two strains of *Salmonella*. The specific cell features which are reflected in this term are under debate, but the magnitude of this measured property will not change (Pethig, 2019). The electric field and the gradient of the electric field combination $\left(\frac{\nabla |\vec{E}{|}^{2}}{E^{2}}\cdot \vec{E}\right)$ are simulated to provide the EKMr for each strain.

## Materials and Methods

### Bacterial Culture and Sample Preparation

sv. *Cubana* (ATCC 12007) and sv. *Poona* (ATCC BAA-1673) were obtained from ATCC. Each strain was grown on triple sugar iron agar for 4 days at ambient temperature. Ten milliliters of sterile 3% tryptic soy broth was inoculated, and the serotype solutions were incubated in a shaker/incubator at 250 rpm (37°C) for 19 h. The concentration of cells is about 10^9^ CFU/mL. The cultures were stored at 4°C.

A volume of 100 μl of each culture was dissolved into 900 μl 5 mM 4-(2-hydroxyethyl)-1-piperazineethanesulfonic acid (HEPES) buffer (pH = 7.3) solution and centrifuged for 5 min at 2000 × *g*. The supernatant was removed, and the washing procedure was repeated three times with HEPES buffer solution. The sample was suspended in 1 ml 5 mM HEPES buffer solution prior to use. Microbial cultures are required at Biosafety Level 1 or 2 or 3. All the experiments were performed with Biosafety Level 2 space and procedures.

### Microdevice Design, Simulation, and Fabrication

A microchannel described in a previous work (Staton et al., 2010) and used for other cellular studies (Jones et al., 2014, 2015; Crowther et al., 2019) was used for the biophysical behavior study of *Salmonella* strains. In brief, opposing pairs of triangles were designed to constitute the sawtooth shape of the channel (Figure 1A). The length between the inlet and outlet of the channel is 4.2 cm. The increasing size of the triangles in the channel restrict the narrowest width of the pathway of each gate from 945 to 27 μm with a depth of 16.9 ± 1 μm. Soft lithography was used to fabricate the microchannels using PDMS (Sylgard 184, Dow/Corning, Midland, MI, United States).

Finite element modeling (COMSOL, Inc., Burlington, MA, United States) of the distribution of the electric field in the microchannel was performed as previously detailed (Crowther and Hayes, 2017). The *AC/DC module* was used to interrogate the$\vec{E},\nabla |\vec{E}{|}^{2}$, and $\frac{\nabla |\vec{E}{|}^{2}}{E^{2}}\cdot \vec{E}$ in an accurately scaled 2D model of the microchannel.

### Experimental Procedure

The microdevice channel was treated with 5% (w/v) bovine serum album in 2 mM phosphate buffer at pH 7.4 and rinsed with 5 mM HEPES buffer solution before introducing the prepared bacterial sample. Dielectrophoresis behaviors of the two strains were observed by an Olympus IX70 inverted microscope with a 4× or 10× objective. Images and videos were recorded by QICAM cooled CCD camera (QImaging, Inc., Surrey, BC) and Streampix III image capture software (Norpix, Inc., Montreal, QC). A voltage between 0 – 3000 V was applied to platinum electrodes (0.404-mm external diameter, 99.9% purity, Alfa Aesar, Ward Hill, MA) connected to the inlet (+) and outlet (ground) to capture and study behaviors of *Salmonella* strains. Analysis and error assessment were based on 4 individual trials for each strain.

## Results

**FIGURE 2:**
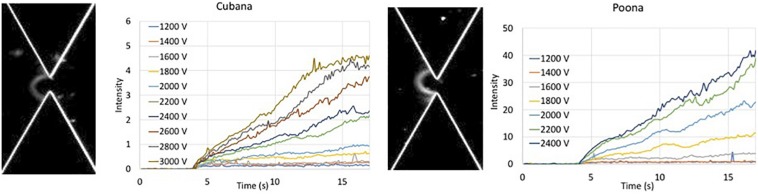
Intensity of collected bolus of *Salmonella* versus time at various applied voltages. Intensity of sv. *Cubana* was recorded at voltages from 1200 to 3000 V. Intensity of sv. *Poona* was recorded at voltages from 1200 to 2400 V. Images are recorded at 20 s of 2400 V for sv. *Cubana* **(Left)** and sv. *Poona* **(Right)**.

The biophysical behavior of sv. *Cubana* and sv. *Poona* were investigated in the DC-iDEP device. Each strain was tested separately in various devices of the same design. Both strains produced a pattern of collected cells appearing as distinct arcs near a 27 μm gate at appropriate applied voltages (Figures 1A, 2; Jones et al., 2014, 2015; Crowther et al., 2019). With 1600 V applied, sv. *Poona* showed capture behavior whereas the same electric field conditions did not capture sv. *Cubana*. The intensity of concentrated sv. *Poona* increases with the time of 1600 V applied at 0, 10, 18, 24 s. The strain dispersed as expected when the electric field was removed, which indicated the effective removal of the EK and DEP forces on the particles. With higher voltages applied, capture of sv. *Cubana* was then observed.

The intensity within the capture area was recorded where the increased intensity reflected the collection of the cells. The intensity curves of sv. *Cubana* from 1200 to 3000 V and sv. *Poona* from 1200 to 2400 V in 200 V increments were plotted (Figure 2). The intensities increase with higher applied voltages for both sv. *Cubana* and sv. *Poona*.

**FIGURE 3:**
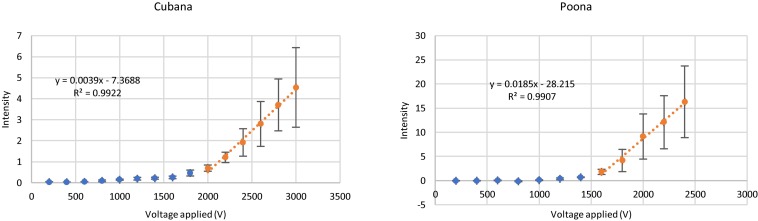
Capture behaviors of sv. *Cubana* and sv. *Poona* at various applied potentials. Intensity was recorded at a 27-μm gate when the potential was applied for 10 s. Error bars represent SEM.

Data were analyzed at a constant time (10 s after voltage applied) for sv. *Cubana* and sv. *Poona* (Figure 3). For the blue data points at lower voltages, where no capture occurred, no significant change of the intensity is observed and is comparable to the background. The orange data points at higher voltages, from 2000 V for sv. *Cubana* and from 1600 V for sv. *Poona*, were used for plotting the linear regression line of the increased intensity reflective of cell accumulation. Error bars reflect standard error of the mean (SEM).

The slope and intercept of the linear fits were used to determine the rate of particle accumulation and onset voltage for capture (Hilton and Hayes, 2019). In this way, initial capture voltages were determined to be 1889 ± 228 V for sv. *Cubana* and 1525 ± 196 V for sv. *Poona*. Using the multiphysics calculations to determine the field and gradient values, the EKMr was determined to be 2.7 ± 0.3 × 10^10^ V/m^2^ for sv. *Cubana* and 2.2 ± 0.3 × 10^10^ V/m^2^ for sv. *Poona*. These are well differentiated and sufficiently different to be considered statistically significant.

**FIGURE 4:**
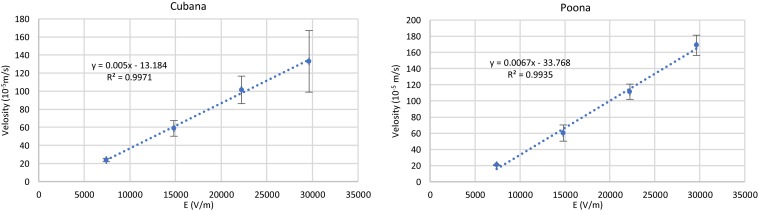
Plots of velocity of sv. *Cubana* and sv. *Poona* with varying electric field to determine μ_EK_. (${\vec{\text{v}}}_{\text{EK}}=\mu_{\text{EK}}\,\vec{E}$). Error bars represent SEM.

The EK behaviors of the strains were determined according to Eq. 6 by particle tracking to monitor the velocity while varying electric field strength (Figure 4). The slopes of the linear fits determine the sv. *Cubana* μ_EK_ to be 5.0 ± 0.5 × 10^–8^ m^2^/Vs and sv. *Poona* μ_EK_ to be 6.7 ± 0.3 × 10^–8^ m^2^/Vs. With EKMr $\left(\frac{\mu_{\text{EK}}}{\mu_{\text{DEP}}}\right)$ and μ_EK_ values, μ_DEP_ of sv. *Cubana* was calculated to be 1.8 ± 0.3 × 10^–18^ m^4^/V^2^s and for sv. *Poona* it was determined to be 3.0 ± 1.3 × 10^–18^ m^4^/V^2^s.

**FIGURE 5:**
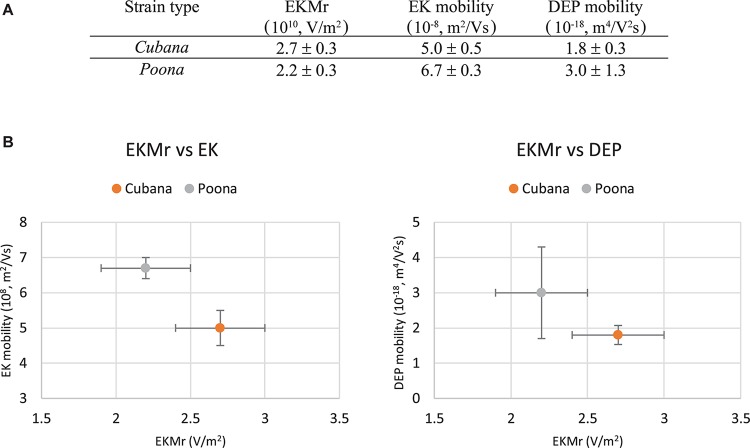
Mobilities of sv. *Cubana* and sv. *Poona* **(A)**. Electrokinetic mobility is determined from velocimetry measurements in the more open parts of the device channel and was used with EKMr to calculate the DEP mobility. The EKMr is plotted versus the EK and DEP mobilities separately **(B)**. The strains are well differentiated by all metrics used. Error bars represent SEM.

The two closely related *Salmonella* strains were differentiated by mobility comparisons (Figure 5). They can be both distinguished by EKMr/EK and EKMr/DEP mobilities. The two strains are demonstrated to have different biophysical behaviors distinguished by the DC-iDEP device.

## Discussion

The sv. *Cubana* and sv. *Poona*. are closely related with regards to the *Kauffmann–White* nomenclature (Figure 1B). According to this classification scheme, there is one known difference in the O Group (*Cubana* – O: 23; *Poona* – O: 22) and several differences in the H Antigens, sv. *Cubana* having z_29_, [z_37_] and [z_49_] and sv. *Poona* expressing z, 1, 6, [z_44_] and [z_59_] (notation details retained from nomenclature guide) (Grimont and Weill, 2007). Even though these serovars are considered “close” with the *Kauffman–White* classification system, there are still clear and identifiable differences in the chemical structure of the surface of the cell which may influence how they interact with an electric field.

Dielectrophoresis and cellular impedance spectroscopy have demonstrated a capability to differentiate cells based upon changes in the biochemical makeup of the cellular structure with labels (Labeed et al., 2003, 2011; Chin et al., 2006; Coley et al., 2007; Flanagan et al., 2008; Jones et al., 2015; Su et al., 2016; Fernandez et al., 2017; Rohani et al., 2018; Crowther et al., 2019; Liu et al., 2019; Hilton et al., 2020). When fully developed and vetted, this approach of serotyping will require less expense and expertise compared to producing antisera in agglutination test utilizing O and H antiserum and will not require expert genomic information interpretation skills in whole genome sequencing for identifying *Salmonella* (Ng and Kirkness, 2010; Ashton et al., 2016).

The relationship between biophysical behaviors, zeta potential, and the mobilities has been described (Hilton and Hayes, 2019). Zeta potential has a linear relationship with EK mobility and results in the difference in the onset voltage and the concentration slope. The differences in the conductivity of the serotypes could also affect the capture onset potential because the change of the DEP mobility. However, the conductivity and permittivity of the medium contribute little to the capture onset potentials for the behaviors of two serotypes but has a significant effect to the accumulation slope.

The EK mobility is significantly different between these strains, supporting the conclusion that the surface charge is changed (Figure 4; Hilton and Hayes, 2019). This a reasonable result since the surface antigens are known to be different between sv. *Cubana* and sv. *Poona.* In addition, the DEP mobility differs between the strains, showing that both surface and interior electrophysical properties differ, although it is impossible to assign a specific ratio to the relative effect from each (Hilton and Hayes, 2019). The biophysical differences between the two strains are reasonable with regards to the biological and biochemical alterations noted in the nomenclature alone, without considering other undocumented effects. The eventual impact of these results is yet to be understood in the serotyping laboratory; it is not known what other techniques will be enhanced by using this as a pre-screening or concentrating tool or if it will eventually develop into a standalone serotyping mechanism for well-known and vetted samples. An interesting question which remains to be answered is whether the magnitude of the differences in biophysical properties have any correlation with the total known and identified differences in the strains.

Previous DC-iDEP work successfully distinguished closely related strains of microbes (Jones et al., 2014, 2015; Crowther et al., 2019; Hilton, 2019; Hilton et al., 2020). The connectivity between biophysical properties and current notions of “relatedness” of two strains is unknown. The biophysical properties relate in a non-linear fashion with traditional cataloging systems [genetics, transcriptome, proteomics, molecular recognition (immune-, selex), and/or metabolic assessments]. In the author’s laboratory, all previously attempted paired biophysical differentiation were successful and include: *Staphylococcus epidermidis* (gentamicin resistant/susceptible) (Jones et al., 2015), various strains of *Escherichia coli* (Jones et al., 2014), *Listeria monocytogenes* strains (Crowther et al., 2019), *S*taphylococcus *aureus* (methicillin resistance/susceptible) (Hilton et al., 2020), and *Klebsiella pneumoniae* (Hilton, 2019). Within the species *Salmonella*, some interesting future works would include the differentiation paired serotypes *Salmonella* typhimurium compared to *Salmonella* typimurium monophasic variant (*Salmonella* 1,4,[5],12:i:-) and *Salmonella* Indiana (O4,12;z;1,7) compared to *Salmonella* Loubomo (O4,12;z;1,6).

In developing and discussing this technique over many venues, some themes emerged which have proven instructive. First is that the effects of biologically important changes may not induce a measurable change in the cells using the electric field effects. The current study undermines this concept, along with many previous quantitative assessments (Jones et al., 2014, 2015; Crowther et al., 2019; Hilton and Hayes, 2019; Liu et al., 2019). The second theme is that the variation in the biological entities will be too great to decipher. Interestingly enough, this high-resolution capability provides a tool to investigate the origins and structure of that variability. Within any population of cells some will have a biologically significant change and others will have routine property variances which are not biological differentiators. This technique can allow for quantitative determination of biophysical-to-biological action connectivity.

## Conclusion

This work demonstrates the differentiation of the two close strains of *Salmonella*, sv. *Cubana* and sv. *Poona*, by DC-iDEP device. The variable capture conditions are accomplished without labels or otherwise altering the cells, the effects occur due to the native condition of the organisms with the setting of electric field properties. These results support the concept that biophysical separation and concentration will potentially become a useful tool in the microbiology laboratory to aid in serotyping of *Salmonella.*

## Data Availability Statement

All datasets generated for this study are included in the article/supplementary material.

## Author Contributions

YL was responsible for data collection, experimental condition testing, and manuscript preparation. MH worked on the overall instruction on the research and manuscript preparation.

## Conflict of Interest

MH is an investor in and a collaborator with Charlot Biosciences, a company which has licensed the underlying technology presented in this paper. The remaining author declares that the research was conducted in the absence of any commercial or financial relationships that could be construed as a potential conflict of interest.
